# Oxidative Stress in Plants

**DOI:** 10.3390/antiox9060481

**Published:** 2020-06-03

**Authors:** Mounira Chaki, Juan C. Begara-Morales, Juan B. Barroso

**Affiliations:** Group of Biochemistry and Cell Signaling in Nitric Oxide, Department of Experimental Biology, Center for Advanced Studies in Olive Grove and Olive Oils, Faculty of Experimental Sciences, University of Jaén, Campus Las Lagunillas, s/n, E-23071 Jaén, Spain; mounira@ujaen.es (M.C.); jbegara@ujaen.es (J.C.B.-M.)

Environmental stresses negatively affect plant growth, development and crop productivity. These adverse conditions alter the metabolism of reactive oxygen and nitrogen species (ROS and RNS, respectively). The high concentrations of these reactive species that exceed the capacity of antioxidant defence enzymes, disturb redox homeostasis, which could trigger damage to macromolecules, such as membrane lipids, proteins and nucleic acids, and ultimately result in nitro-oxidative stress and plant cell death. Significant progress has been made to understand how plants persist in these stressful environments that could be vital to improve plant crop yield. In this special issue “*Oxidative Stress in Plants*”, both original articles and detailed reviews have been published with the aim to provide an up-date view in this research area in higher plants. 

In the natural environment, plants are constantly exposed to abiotic stresses, such as extreme temperatures, salt stress, drought and heavy metals that have a huge impact on agriculture worldwide and consequently, lead to massive economic losses. In this sense, three research papers have analysed the effect of abiotic stress on plant growth and development. Dr. Wani’s group [1] studied the role of *Serratia marcescens* BM1 in response to cadmium (Cd) stress in soybean plants by different physiological, biochemical and molecular assays. They found that, in Cd-stressed plants, the *Serratia marcescens* BM1 treatment not only down-regulated Cd levels and oxidative stress markers, but also up-regulated levels of osmolytes, stress-related gene expression and activities of antioxidant enzymes. These authors suggested that inoculation with the *Serratia marcescens* BM1 would promotes Cd stress tolerance and phytoremediation potential. The impact of abiotic stress was also reported by Dr. Barroso’s group [2] as they demonstrated the effect of short-term low temperature stress on the metabolism of reactive oxygen and nitrogen species in *Arabidopsis* plants. These authors showed that the low temperature produces nitro-oxidative stress, and reduces cytosolic NADP-malic enzyme activity, which was negatively modulated by the protein tyrosine nitration process. In addition, they proposed that Tyr73 would be a possible residue to be involved in reducing this enzymatic activity. Moreover, Dr. Rivero’s group [3] investigated the response of tomato plants to the effects of calcium and potassium on plant tolerance to combined high-temperature and salinity stress conditions. They showed the positive effect of a rise in calcium and potassium in the nutrient medium on the improvement of oxidative stress produced under these environmental stress injuries. The authors underlined the importance of the correctly administering of nutrient solution and fertilisation to face the damaging effects of adverse conditions in plant cells.

On the other hand, plant cells develop an antioxidant defence mechanism, which includes the non-enzymatic and enzymatic antioxidants for the detoxification of ROS. However, if the ROS production is higher than the ability of the antioxidant systems to scavenge them, it can lead to oxidative stress, and finally to cell death. In this context, Dr. De Maio’s group [4] used citrus plants to investigate the modulation of poly (ADP-ribose) polymerase and antioxidant enzymes, using leaves in different developmental stages, including young, mature and senescent. Their work addressed the physiological, biochemical and molecular changes that occur in plant cells during leaf ageing. In young leaves, photochemical and glutathione-S-transferase activities increased. However, while the ageing process advanced, the non-enzymatic antioxidant systems reduced and reached the lowest levels in senescent leaves, while poly (ADP-ribose) polymerase activity increased. In the same way, Hasanuzzaman et al. [5] discussed in an extensive review, the available and up-to-date knowledge on the Ascorbate-Glutathione pathway concerning the oxidative stress tolerance, as well as plant defence mechanisms. Furthermore, the review by Laxa et al. [6] provided up-to-date information about the response and function of ROS and RNS, mainly with regard to superoxide radicals, hydrogen peroxide and nitric oxide under drought stress conditions, and their scavenging by the antioxidant defence enzymes in several plant species. To better understand the interaction between chitosan and *Vitis vinifera* L. plants, the original article by Singh et al. [7] analysed the antioxidant potential, the total phenolic content and the expression of ROS detoxification genes in two red grapevine varieties treated by chitosan. They concluded that chitosan induced the phenolic compounds, as well as acted as the organiser for the transfer of polyphenols from the *Vitis vinifera* leaves to the berries.

Another interesting feature of this special issue focuses on investigating the other H_2_O_2_ targets involved in programmed cell death. Dr. Mano’s group [8] studied the mechanism that increased the reactive carbonyl species in the H_2_O_2_-produced programmed cell death in tobacco Bright Yellow-2 cells. They suggested that H_2_O_2_ initially inactivates a carbonyl reductase(s), which increases the reactive carbonyl species content, leading to the activation of the caspase-3-like protease of the 20S proteasome. The authors proposed that carbonyl reductase acted as a ROS sensor for inducing programmed cell death. 

In plant cells, the ROS metabolism has been widely studied in different compartments, including mitochondria, cytosol, chloroplast, cell wall, plasma membrane, apoplast, glyoxysomes and peroxisomes [9]. The review by Dr. Petřivalský’s group [10] provided the present knowledge about the compartment-specific pathways of reactive oxygen species generation and decomposition in plant cells, and the mechanisms that controlling their homeostasis in cell compartments. Likewise, with a particular example at the chloroplastic level, in an in-depth review Miyake [11] summarised the current research concerning the molecular mechanisms of ROS formation and suppression in photosystem I. He established a novel molecular mechanism for the oxidation of the P700 oxidation system in photosystem I and the elimination of ROS formation from the strong relationship between the light and dark reactions of photosynthesis. Furthermore, in an original article, Lewandowska et al. [12] investigated the effect of H_2_O_2_ on the structure and function of Arabidopsis chloroplastic DJ-1B. They found that AtDJ-1B has double functions, namely holdase and glyoxalase activity, which responded differently to H_2_O_2_. Glyoxalase activity was reduced by H_2_O_2_, however the holdase chaperone function did not change. They also analysed the phenotype of T-DNA lines that lacked the protein, and showed that AtDJ-1B was not necessary for plant growth under stress stimuli.

In summary, to better understand the nitro-oxidative stress networks in higher plants (Figure 1), the subjects addressed in this special issue provide an update and new knowledge about ROS and RNS metabolisms in plant responses to adverse environmental stimuli and the modulation of antioxidant systems to control ROS production and accumulation.

## Figures and Tables

**Figure 1:**
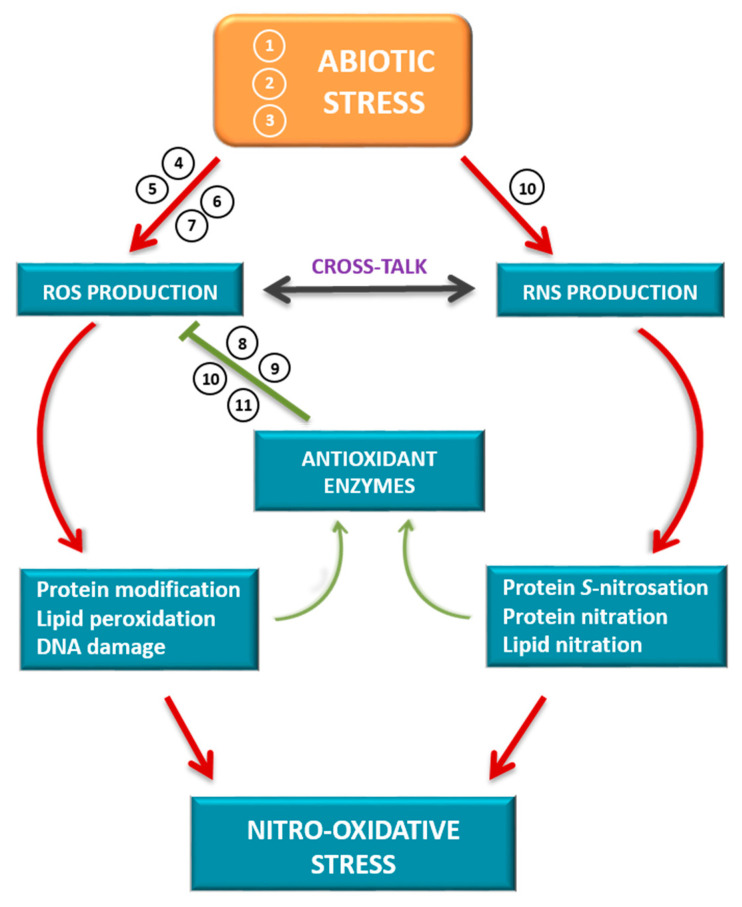
Schematic model of cross-talk between reactive oxygen species (ROS) and reactive nitrogen species (RNS) in plant responses to abiotic stress. Different abiotic stress situations can generate an uncontrolled production of ROS and RNS that oxidatively modify different biomolecules (proteins, lipids and nucleic acids). These modifications can lead to a gain of function of the antioxidant systems to control the production of ROS or generate a situation of cellular damage supported by a process of nitro-oxidative stress. The numbers indicate the relationship of each article in the Special Issue to the subject matter covered. (1) El-Esawi et al., 2020 [1]. (2) Begara-Morales et al., 2019 [2]. (3) García-Martí et al., 2019 [3]. (4) Biswas et al., 2020 [8]. (5) Janků et al., 2019 [10]. (6) Miyake, 2020 [11]. (7) Lewandowska et al., 2019 [12]. (8) Arena et al., 2019 [4]. (9) Hasanuzzaman et al., 2019 [5]. (10) Laxa et al., 2019 [6]. (11) Singh et al., 2019 [7].

## References

[1] El-Esawi M.A., Elkelish A., Soliman M., Elansary H.O., Zaid A., Wani S.H. (2020). *Serratia marcescens* BM1 Enhances Cadmium Stress Tolerance and Phytoremediation Potential of Soybean Through Modulation of Osmolytes, Leaf Gas Exchange, Antioxidant Machinery, and Stress-Responsive Genes Expression. Antioxidants.

[2] Begara-Morales J.C., Sánchez-Calvo B., Gómez-Rodríguez M.V., Chaki M., Valderrama R., Mata-Pérez C., López-Jaramillo J., Corpas F.J., Barroso J.B. (2019). Short-Term Low Temperature Induces Nitro-Oxidative Stress that Deregulates the NADP-Malic Enzyme Function by Tyrosine Nitration in *Arabidopsis thaliana*. Antioxidants.

[3] García-Martí M., Piñero M.C., García-Sanchez F., Mestre T.C., López-Delacalle M., Martínez V., Rivero R.M. (2019). Amelioration of the Oxidative Stress Generated by Simple or Combined Abiotic Stress through the K^+^ and Ca^2+^ Supplementation in Tomato Plants. Antioxidants.

[4] Arena C., Vitale L., Bianchi A.R., Mistretta C., Vitale E., Parisi C., Guerriero G., Magliulo V., De Maio A. (2019). The Ageing Process Affects the Antioxidant Defences and the Poly (ADPribosyl)ation Activity in *Cistus Incanus* L. Leaves. Antioxidants.

[5] Hasanuzzaman M., Bhuyan M.H.M.B., Anee T.I., Parvin K., Nahar K., Mahmud J.A., Fujita M. (2019). Regulation of Ascorbate-Glutathione Pathway in Mitigating Oxidative Damage in Plants under Abiotic Stress. Antioxidants.

[6] Laxa M., Liebthal M., Telman W., Chibani K., Dietz K.-J. (2019). The Role of the Plant Antioxidant System in Drought Tolerance. Antioxidants.

[7] Singh R.K., Soares B., Goufo P., Castro I., Cosme F., Pinto-Sintra A.L., Inês A., Oliveira A.A., Falco V. (2019). Chitosan Upregulates the Genes of the ROS Pathway and Enhances the Antioxidant Potential of Grape (*Vitis vinifera* L. ‘Touriga Franca’ and ’Tinto Cão’) Tissues. Antioxidants.

[8] Biswas M.S., Terada R., Mano J. (2020). Inactivation of Carbonyl-Detoxifying Enzymes by H_2_O_2_ Is a Trigger to Increase Carbonyl Load for Initiating Programmed Cell Death in Plants. Antioxidants.

[9] Mignolet-Spruyt L., Xu E., Idänheimo N., Hoeberichts F.A., Mühlenbock P., Brosché M., Van Breusegem F., Kangasjärvi J. (2016). Spreading the news: Subcellular and organellar reactive oxygen species production and signalling. J. Exp. Bot..

[10] Janků M., Luhová L., Petřivalský M. (2019). On the Origin and Fate of Reactive Oxygen Species in Plant Cell Compartments. Antioxidants.

[11] Miyake C. (2020). Molecular Mechanism of Oxidation of P700 and Suppression of ROS Production in Photosystem I in Response to Electron-Sink Limitations in C3 Plants. Antioxidants.

[12] Lewandowska A., Vo T.N., Nguyen T.-D.H., Wahni K., Vertommen D., Van Breusegem F., Young D., Messens J. (2019). Bifunctional Chloroplastic DJ-1B from *Arabidopsis thaliana* is an Oxidation-Robust Holdase and a Glyoxalase Sensitive to H_2_O_2_. Antioxidants.

